# Complete genome sequence of the thermophilic *Acidobacteria*, *Pyrinomonas methylaliphatogenes* type strain K22^T^

**DOI:** 10.1186/s40793-015-0099-5

**Published:** 2015-11-14

**Authors:** Kevin C. Y. Lee, Xochitl C. Morgan, Jean F. Power, Peter F. Dunfield, Curtis Huttenhower, Matthew B. Stott

**Affiliations:** GNS Science, Extremophiles Research Group, Taupō, New Zealand; Department of Biostatistics, Harvard School of Public Health, Boston, MA USA; Broad Institute of Harvard and MIT, Cambridge, MA USA; Department of Biological Sciences, University of Calgary, Calgary, Canada

**Keywords:** *Acidobacteria*, *Pyrinomonas*, New Zealand, Thermophile, Soil, Geothermal

## Abstract

Strain K22^T^ is the type species of the recently- described genus *Pyrinomonas*, in subdivision 4 of the phylum *Acidobacteria* (Int J Syst Evol Micr. 2014; 64(1):220–7). It was isolated from geothermally-heated soil from Mt. Ngauruhoe, New Zealand, using low-nutrient medium. *P. methylaliphatogenes* K22^T^ has a chemoheterotrophic metabolism; it can hydrolyze a limited range of simple carbohydrates and polypeptides. Its cell membrane is dominated by *iso-*branching fatty acids, and up to 40 % of its lipid content is membrane-spanning and ether lipids. It is obligately aerobic, thermophilic, moderately acidophilic, and non-spore-forming. The 3,788,560 bp genome of *P. methylaliphatogenes* K22^T^ has a G + C content of 59.36 % and contains 3,189 protein-encoding and 55 non-coding RNA genes. Genomic analysis was consistent with nutritional requirements; in particular, the identified transporter classes reflect the oligotrophic nature of this strain.

**Electronic supplementary material:**

The online version of this article (doi:10.1186/s40793-015-0099-5) contains supplementary material, which is available to authorized users.

## Introduction

Phylotypes from the phylum *Acidobacteria*^1^ are commonly detected across a range of ecosystems, including marine and freshwater bodies, sediments, geothermal systems, and soils. Despite the apparent ubiquitous distribution acidobacterial phyotypes, particularly in soil environments, only 17 acidobacterial genera (represented by formal description and publication of respective type strains, in accordance with the International Code of Nomenclature of Prokaryotes [1]) have been validly published [2, 3]. Here we present a description of the complete genome sequence and annotation of *Pyrinomonas methylaliphatogenes* strain K22^T^ (= DSM 25857 = ICMP 18710), the type species of the genus *Pyrinomonas* within subdivision 4 of *Acidobacteria*.

*Pyrinomonas methylaliphatogenes* K22^T^ was isolated from a fumarole on the outer crater rim of the stratovolcano Mt. Ngauruhoe [4]. It exhibits a Gram-negative cell wall, is non-spore-forming, and is catalase- and oxidase-positive (Table 1). It is a thermophilic and moderately acidophilic obligately aerobic chemoorganotroph. Of particular note is its unusual lipid composition that is dominated by odd-numbered saturated *iso-*branching fatty acids (*iso-*C_15:0_, *iso-*C_17:0_, *iso-*C_19:0_ and *iso-*C_21:0_ that total >88.5 % of the total fatty acid extract) [4]. In addition, >40 % of the total membrane lipid content is made up by *iso-*branching glycerol ether analogues of the cellular fatty acids and membrane-spanning *iso-*diabolic acids [5]. Membrane-spanning and ether lipids occur ubiquitously in *Archaea*, but in recent studies have also been commonly detected in cultivated representatives in subdivision groups 1, 3 and 4 of *Acidobacteria* [5, 6].

**Table 1:** Classification and general features of *P. methylaliphatogenes* K22^T^

MIGS ID	Property	Term	Evidence code^a^
	Current classification	Domain *Bacteria*	TAS [35]
		Phylum *Acidobacteria*	TAS [36]
		Class ‘Insertae sedis 99’	
		Order ‘Insertae sedis 100’	
		Family ‘Insertae sedis 101’	
		Genus *Pyrinomonas*	TAS [4]
		Species *Pyrinomonas methylaliphatogenes*	TAS [4]
		Type strain K22^T^ (=DSM 25857^T^ =ICMP 18710^T^).	TAS [4]
	Gram stain	negative	TAS [4]
	Cell shape	rod	TAS [4]
	Motility	non-motile	TAS [4]
	Sporulation	non-sporulating	TAS [4]
	Temperature range	thermophilic (50–69 °C)	TAS [4]
	Optimum temperature	65 °C	TAS [4]
	pH range	moderately acidophilic (4.1–7.8)	
	Optimum pH	6.5	
	Carbon source	peptides, proteins, carbohydrates	TAS [4]
	Terminal electron receptor	oxygen	TAS [4]
	Energy metabolism	chemoorganotroph	TAS [4]
MIGS-6	Habitat	geothermal soil	TAS [37]
MIGS-6.3	Salinity	non-halophile (no growth > 1 % (w/v) NaCl)	TAS [4]
MIGS-22	Oxygen requirement	obligate aerobe	TAS [4]
MIGS-15	Biotic relationship	free-living	TAS [4]
MIGS-14	Pathogenicity	not reported	NAS
MIGS-4	Geographic location	Mt Ngauruhoe, New Zealand	TAS [37]
MIGS-5	Sample collection	2006	NAS
MIGS-4.1 MIGS-4.2	Latitude – Longitude	39° 9’25.31”S - 175° 38’6.74”E	IDA
MIGS-4.3	Depth	not reported	IDA
MIGS-4.4	Altitude	2,270 m	IDA

^a^Evidence codes - *IDA* inferred from direct assay, *TAS* traceable author statement (i.e., a direct report exists in the literature), *NAS* non-traceable author statement (i.e., not directly observed for the living, isolated sample, but based on a generally accepted property for the species, or anecdotal evidence). These evidence codes are from the Gene Ontology project [38]

Subdivision 4 of the *Acidobacteria* has five validly-named species: *P. methylaliphatogenes* K22^T^,[4] *Chloracidobacterium thermophilum* [7, 8], *Blastocatella fastidiosa* [9], *Aridibacter famidurans*, and *Aridibacter kavangonensis* [3]. The latter three species are phylogenetically distant from *P. methylaliphatogenes* K22^T^, are mesophilic and have differing pH ranges and substrate utilization profiles from that of *P. methylaliphatogenes* K22^T^. *Chloracidobacterium thermophilum* is a moderately thermophilic facultatively anoxygenic photoheterotroph isolated from a hotspring microbial mat at Yellowstone National Park [7, 8]. An additional strain, Ellin6075 was isolated from an Australian pasture soil, and is a mesophilic heterotroph that derives its energy from complex carbohydrate sources, but has little information available regarding its phenotypic traits [10]. Common features shared by subdivision 4 strains include an aerobic and heterotrophic phenotype [3, 4], and membrane lipid *iso*-diabolic acids [5].

## Organism information

### Classification and features

**Fig. 1:**
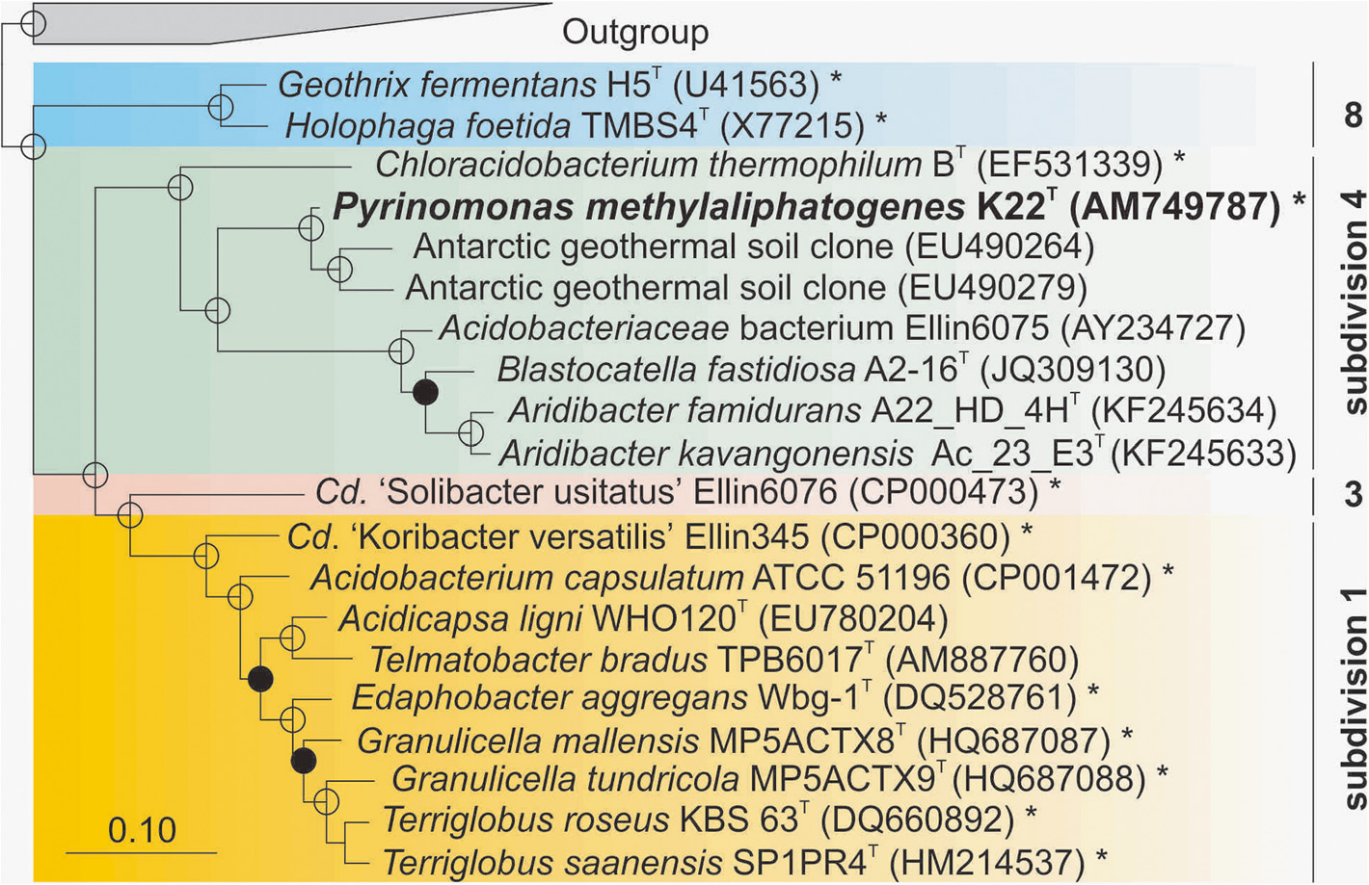
Phylogenetic tree based on 16S rRNA gene sequences of *Pyrinomonas methylaliphatogenes* K22^T^ (highlighted) and other cultivated strains and clonal phylotypes within the phylum *Acidobacteria*. Four of the acidobacterial subdivisions are included. The tree was constructed via a Bayesian inference model (MrBayes), using Markov Chain Monte Carlo (MCMC - 2,000,000 resamples, four chains, temperature = 0.5) sampling methods to calculate posterior distributions of trees in the ARB software environment. Posterior probability values ≥ 90 % are indicated by open circles, ≥80 % by filled circles, and ≥70 % by open diamonds. The scale bar represents a 0.1 change per nucleotide position. Strains whose genomes have been sequenced, are marked with an asterisk; *G. fermentans* H5^T^ (NZ_AUAU00000000), *H. foetida* TMBS4^T^ (AGSB00000000), *C. thermophilum* B^T^ (CP002414), *P. methylaliphatogenes* K22^T^ (CBXV000000000), *Candidatus* ‘S. usitatus’ Ellin6076 (CP000473), *Candidatus* ‘K. versatilis’ Ellin345 (CP000360), *Acidobacterium capsulatum* ATCC 51196^T^ (CP001472), *Edaphobacter aggregans* Wbg-1^T^ (JQKI00000000), *Granulicella mallensis* MP5ACTX9^T^ (CP003130), *Granulicella tundricola* MP5ACTX9^T^ (CP002480), *Terriglobus roseus* KBS63^T^ (CP003379), and *Terriglobus saanensis* SP1PR4^T^ (CP002467). The phylotypes strains used as an outgroup included *Thermoanaerobaculum aquaticum* MP-01^T^ (JX4200244), *Dictyoglomus thermophilum* H-6-12^T^ (X69194), *Caldisericum exile* AZM16c01^T^ (AB428365), *Hydrogenobacter hydrogenophilus* Z-829^T^ (Z30424), *Thermodesulfobacterium thermophilum* DSM 1276^T^ (AF334601), *Deinococcus roseus* TDMA-uv51 (AB264136), *Truepera radiovicrix* RQ-24^T^ (DQ022076), *Thermus aquaticus* YT-1 (L09663), and *Thermus scotoductus* SE-1^T^ (AF032127)

Phylogenetic distances of closest-related phylotypes and cultivated subdivision 4 acidobacterial strains were determined by aligning the representative near full length 16S rRNA gene sequences (all sequences were > 1,400 nucleotides in length) and calculating sequence similarity via a pair-wise alignment within the ARB software environment [11]. Analysis showed that the 16S rRNA gene sequence of *P. methylaliphatogenes* K22^T^ (AM749787) is 85 % similar to *B. fastidiosa* strain A2-16^T^ (JQ309130), and is 84 % similar to both *A. famidurans* strain A22_HD_4H^T^ (KF245634), and *A. kavangonensis* Ac_23_E3^T^ (KF245633) [3, 4, 9]. In addition, *P. methylaliphatogenes* K22^T^ shares 85 % 16S rRNA gene sequence similarity with both Ellin6075 (AY234727) [7] and *C. thermophilum* B^T^ (EF531339) [8]. The most closely-related phylotypes to *P. methylaliphatogenes* K22^T^ are two sequences from clonal libraries of environmental 16S rRNA genes (EU490264, EU490279) retrieved from geothermal soils on Mt. Erebus, Antarctica [12]; both of these shared 95 % 16S rRNA gene sequence similarity with *P. methylaliphatogenes* K22^T^. Phylogenetic comparison (Fig. 1) showed that *P. methylaliphatogenes* K22^T^ is a taxonomically-distinct genus and species of subdivision 4 in the phylum *Acidobacteria*.

**Fig. 2:**
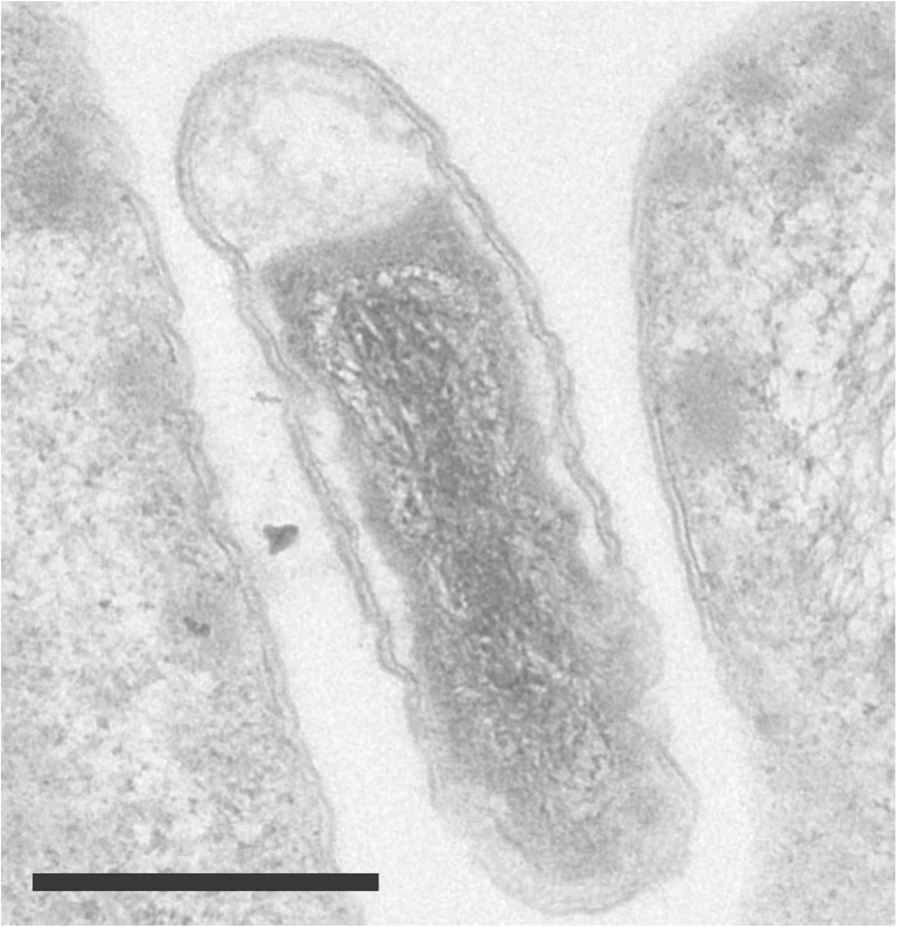
Transmission electron micrograph of *P. methylaliphatogenes* K22^T^ cultured in R2A liquid medium (60 °C), using a Zeiss LEO 912 Energy-Filtering TEM [34]. The scale bar represents 500 nm

*Pyrinomonas methylaliphatogenes* K22^T^ is non-motile and exhibits straight or bent rod cell morphology (0.3 – 0.6 μm in diameter and 1–4 μm in length) (Fig. 2). It has a temperature range (optimum) for growth of 50–69 °C (65 °C) and a pH range (optimum) of 4.1–7.8 (6.5). The bacterium has an obligately aerobic metabolism and can utilize a small selection of simple carbohydrates including glucose, lactate, alginate, mannose, xanthan, xylan, xylose, arabinose, and sucrose, as well as a limited variety of proteinaceous substrates including casamino acids, peptone, tryptone, yeast extract and nutrient broth (Table 1). It obtains nitrogen via the uptake of NO_3_^−^, NH_4_^+^, urea, yeast extract and casamino acids but cannot fix dinitrogen gas. The strain is not able to grow via photosynthesis, nor is it able grow autotrophically using CO_2_ as the sole source of carbon. However, optical density of culture is improved via the provision of additional CO_2_ in the headspace during heterotrophic growth, suggesting an assistive anapleurotic mechanism [4].

#### Chemotaxonomic data

The primary cellular fatty acids are *iso-*C_15:0_ (40.8 %), *iso-*C_17:0_ (30.8 %), *iso-*C_19:0_ (12.1 %) and *iso-*C_21:0_ (4.8 %). *P. methylaliphatogenes* K22^T^ also possesses membrane-spanning dicarboxylic acid 13,16-dimethyl octacosanedioic (*iso-*diabolic) acid and glyceryl ethers of alkyl analogues of *iso-*C_15:0_ and *iso-*C_17:0_ and *iso-*diabolic acid. Its primary cellular quinone is MK-8 and its primary cellular lipids are phosphatidylethanolamine and phosphatidylcholine [4].

## Genome sequencing information

### Genome project history

The genome of *P. methylaliphatogenes* K22^T^ was selected for sequencing on the basis of its phylogenetic position and phenotypic dissimilarity to other cultured *Acidobacteria* strains. The quality draft (QD) assembly and annotation was completed in December 2013. The genome project is deposited in the Genomes OnLine Database Gp0050834. A summary of the project information is shown in Table 2. The EMBL-Bank project accession number is CBXV000000000 and consists of 16 scaffolds. Table 2 presents the project information and its association with MIGS version 2.0 compliance [13].

**Table 2:** Project information

MIGS ID	Property	Term
MIGS-31	Finishing quality	High quality draft
MIGS-28	Libraries used	Two libraries used: One 454 library, one Illumina PE library
MIGS-29	Sequencing platforms	454 GS Junior Titanium, Illumina MiSeq
MIGS-31.2	Fold coverage	75.0 ×
MIGS-30	Assemblers	MIRA 4.0rc2
MIGS-32	Gene calling method	Prodigal
	Locus tag	PYK22
	EMBL ID	CBXV000000000
	EMBL Date of Release	12 January 2015
	GOLD ID	Gp0050834
	BIOPROJECT	PRJEB4906
MIGS-13	Source Material Identifier	DSMZ *DSM 25857*, ICMP *ICMP 18710*
	Project relevance	Microbial diversity of the Taupō Volcanic Zone, Tree of Life

### Growth conditions and genomic DNA preparation

*Pyrinomonas methylaliphatogenes* K22^T^ was grown in 2 × 500 ml volumes of R2A liquid medium [14] at 60 °C in an air headspace (1 : 1 ratio of headspace to medium). The medium was sterilized at 121 °C (15 min, 15 psi) prior to inoculation. After three days of incubation, cells were collected via centrifugation. Culture purity was confirmed using an RFLP digestion (*EcoR1*) of the 16S rRNA gene PCR amplification product (amplification used the 9f/1492r primer set) [4]. The restriction digest pattern was identical to known axenic cultures of *P. methylaliphatogenes* K22^T^. Genomic DNA was extracted from the wet biomass (200 mg) using the Nucleospin for Tissue extraction kit as per the manufacturer’s instructions (Macherey Nagel). The gDNA extract was purified via electrophoresis on a 0.8 % (w/v) agarose gel. The gel extracts were cleaned using a Gel Purification kit as per the manufacturer’s instructions (Macherey Nagel), giving a final concentration of 595 ng 100 μl^−1^. The purified gDNA was then frozen at −20 °C until sequenced.

### Genome sequencing and assembly

Genomic sequencing was conducted using a combination of the Illumina MiSeq and 454 GS Junior platforms. A single-end 454 library was constructed according to the protocols of 454 GS FLX Titanium Rapid Library kits and GS Junior Titanium emPCR kits (Additional file 1). The sequencing of the 454 library yielded 75,215 reads with an average length of 492 bps. The paired-end Illumina library was constructed using the Nextera XT DNA Sample Preparation kit (Illumina), according to the manufacturer's protocol (Additional file 1), and sequenced on a MiSeq (2 × 150 bp paired-end reads), yielding 1,196,578 reads. The combined 454 (28.9 Mbp) and Illumina (301 Mbp) sequencing data were assembled together using the hybrid assembly capability of MIRA 4.0 rc4 [15] (parameter and methodologies provided in Additional file 1). The resulting contigs were manually curated via the Staden package [16], generating scaffolds with an average 75 × coverage. Scaffolds with average coverage two standard deviations below the aforementioned overall genome average were discarded (i.e. 32.5 × coverage threshold). The resulting 16 scaffolds contained 2,302,690 assembled reads and 3188 protein coding genes. The abundance of clustered regularly interspaced short palindromic repeats (CRISPRs) and other repeating elements (e.g. transposons and RHS repeat-encoded genes) may have contributed to the scaffolds junctions, such as those observed in scaffold CBXV010000001, CBXV010000004, CBXV010000005, and CBXV010000006.

### Genome annotation

Genome annotation was processed via the DOE-JGI Integrated Microbial Genome – Expert Review (IMG-ER) annotation pipeline [17] using the following steps/components: Coding sequences (CDSs) were predicted using Prodigal [18]. The predicted CDSs were translated and used to search the National Center for Biotechnology Information (NCBI) non-redundant database, UniProt, TIGRFam, Pfam, PRIAM, KEGG, COG, and InterPro databases. These data sources were combined to ascribe descriptions of the protein tRNAScan-SE tool [19] was used to find tRNA genes, whereas ribosomal RNAs were found by searching against models of the ribosomal RNA genes built from SILVA. Other non-coding RNA such as the RNA components of the protein secretion complex and the RNaseP were identified by searching the genome for the corresponding Rfam profiles using INFERNAL [20]. Transmembrane helices and signal peptide cleavage sites within the putative proteins were predicted via TMHMM [21], and SignalP [22] respectively. Additional annotation and gene function prediction as well as data visualization was conducted within the IMG-ER system [23].

## Genome properties

**Fig. 3:**
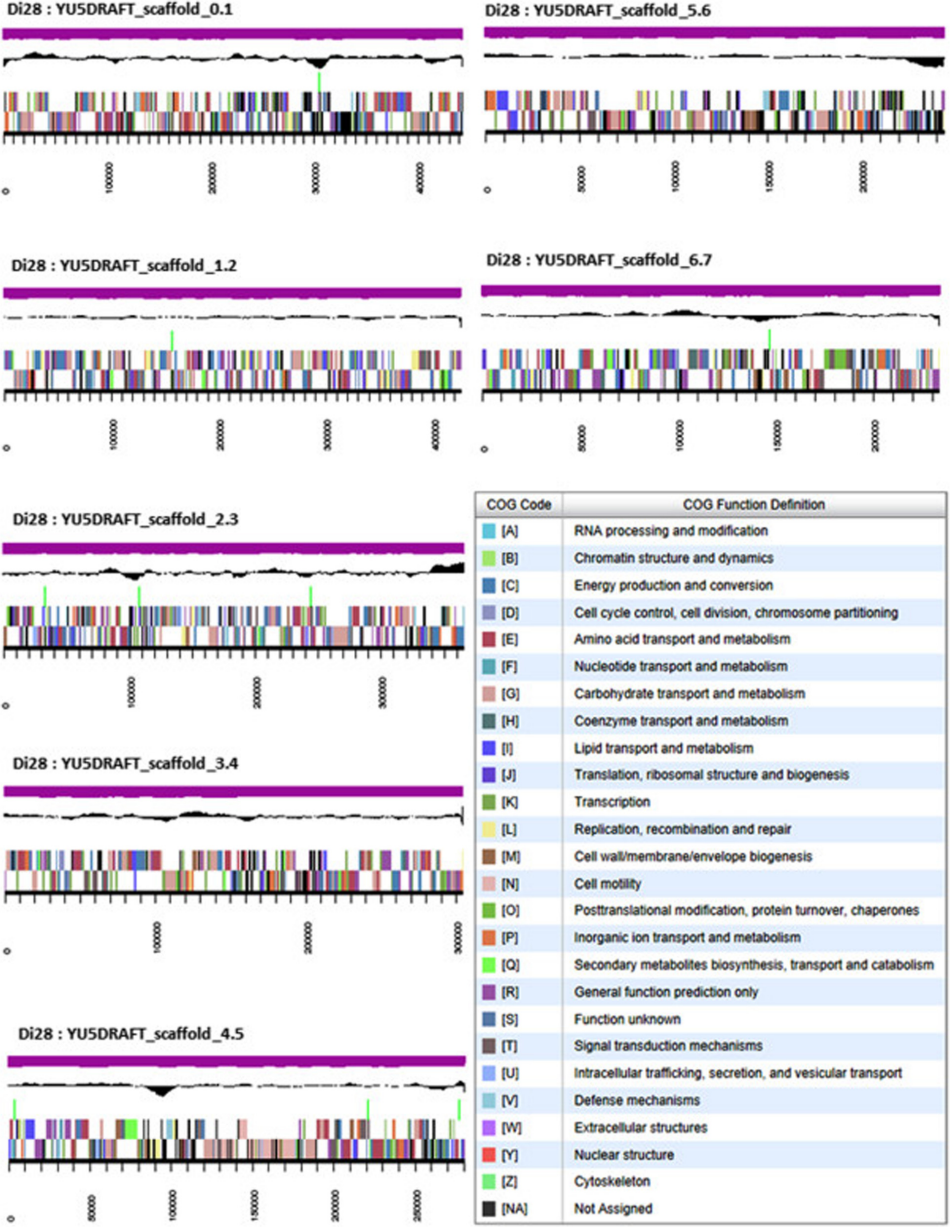
Graphical map of the genome of *P. methylaliphatogenes* K22^T^ showing the eight largest scaffolds. From bottom to the top of each scaffold: Genes on forward strand (color by COG categories as denoted by the IMG platform), genes on the reverse strand (color by COG categories), RNA genes (tRNAs – green, sRNAs – red, other RNAs – black), GC content, and GC skew

The QD assembly of the genome consists of 16 scaffolds totaling 3,788,560 bp in length (59.36 % GC content). Of the 3,244 genes predicted, 3,189 were protein-coding genes, and 55 were non-coding RNA genes. A majority (79.0 %) of genes were assigned putative functions, and the remainder were annotated as hypothetical proteins. The properties and the statistics of the *P. methylaliphatogenes* K22^T^ genome and the distribution of genes into COG functional categories are presented in Table 3, Table 4, and Fig. 3.

**Table 3:** Genome statistics

Attribute	Genome (total)	
	Value	% of total^a^
Size (bp)	3,788,560	100.0
DNA coding (bp)	3,353,298	88.5
G + C content (bp)	2,249,198	59.36
DNA Scaffolds	16	
Total genes^b^	3,244	100.00
Protein-coding genes	3,189	98.3
RNA genes	55	1.7
Pseudo genes	0	0.0
Genes in paralog clusters	2535	78.4
Protein coding genes with function prediction	2,564	79.0
Genes assigned to COGs	2,023	62.3
Genes assigned Pfam domain	2,605	80.3
Genes with signal peptides	293	9.0
Genes with transmembrane helices	766	23.7
CRISPR repeats	15	

^a^The percentage total is based on either the size of the genome in base pairs or the total number of protein coding genes in the annotated genome

**Table 4:** Number of genes associated with the general COG functional categories

Code	Value	% of total^a^	Description
J	137	5.01	Translation, ribosomal structure and biogenesis
A	1	0.03	RNA processing and modification
K	103	3.23	Transcription
L	77	2.41	Replication, recombination and repair
B	2	0.06	Chromatin structure and dynamics
D	27	0.85	Cell cycle control, cell division, chromosome partitioning
V	65	2.04	Defense mechanisms
T	101	3.17	Signal transduction mechanisms
M	191	5.99	Cell wall/membrane/envelope biogenesis
N	67	2.10	Cell motility
U	32	1.00	Intracellular trafficking and secretion
O	123	3.85	Posttranslational modification, protein turnover, chaperones
C	127	3.98	Energy production and conversion
G	171	5.36	Carbohydrate transport and metabolism
E	202	6.33	Amino acid transport and metabolism
F	65	2.04	Nucleotide transport and metabolism
H	126	3.95	Coenzyme transport and metabolism
I	105	3.29	Lipid transport and metabolism
P	105	3.29	Inorganic ion transport and metabolism
Q	64	2.01	Secondary metabolites biosynthesis, transport and catabolism
R	218	6.83	General function prediction only
S	85	2.66	Function unknown
-	1,223	38.33	Not in COGs

^a^The total is based on the total number of protein coding genes (3180) in the annotated genome

## Insights from the genome sequence

The *P. methylaliphatogenes* K22^T^ genome assembly has a size of 3.79 Mb with a %G + C content of 59.3, both of which are comparable with the genomes of other sequenced *Acidobacteria* [24]. It possesses complete citric acid and pentose phosphate cycles. A complete electron transport pathway with an F-type ATPase, NADH dehydrogenase and cytochrome C complex, and the presence of genes encoding superoxide dismutase (PYK22_00483-00484) and catalase (PYK22_02691) are consistent with the observed aerobic phenotype. Genes encoding outer membrane secretion (for example, a type II secretion system, PYK22_02507-02511) and protein assembly (Bam complex, PYK22_02371 & 01777) are present, confirming the observed Gram-negative cell wall structure [4]. Interestingly, *P. methylaliphatogenes* K22^T^ possesses a near-complete complement of flagella encoding-genes (possibly missing the proximal rod *flgF* gene) despite having no observed motility. Key genes for all autotrophic carbon fixation pathways were absent. However, it was previously noted that while *P. methylaliphatogenes* K22^T^ was unable to fix carbon, additional CO_2_ to the headspace while growing heterotrophically improved growth [4]. The presence of phosphoenolpyruvate carboxylase and isocitrate dehydrogenase confirmed the ability of *P. methylaliphatogenes* K22^T^ to supplement carbon anapleurotically. No genes encoding the ability to fix dinitrogen gas were found, again confirming previous phenotypic observations. Interestingly, the genome contains a gene cluster encoding a group 5-type [NiFe] hydrogenase (PYK22_03058-03084) similar to that found in *Mycobacterium smegmatis* [25]; this may confer an ability to oxidize tropospheric concentrations of hydrogen for cell maintenance.

Previous phenotypic characterization of *P. methylaliphatogenes* K22^T^ indicated that it possessed a heterotrophic phenotype with the ability to grow on a range of simple carbohydrates. The *P. methylaliphatogenes* K22^T^ genome encodes for a large number of beta-glucosidase and exoglucanase-acting glycosyl hydrolases, reflecting its ability to grow on primarily simple oligosaccharides such as cellobiose, sucrose, and maltose. A single C6 endoglucanase-acting glycosyl hydrolase (PYK22_03181) was identified in the genome despite having no reported growth on complex or crystalline cellulose as energy sources [4]. Two endo-1,4-beta-xylanases genes confer an ability to grow on xylan and xanthan gum.

Transporters encoded in the *P. methylaliphatogenes* K22^T^ genome mainly belong to the ABC-type transporter superfamily and the major facilitator superfamily. This is consistent with previous study of acidobacterial genomes, which suggest these transporters types were adapted for low-nutrient conditions [26]. ABC transporters in *P. methylaliphatogenes* K22^T^ appear to be involved in the transport of carbohydrates (and derivatives) such as ribose, D-xylose, lipopolysaccharide (*rfbAB*, e.g. PYK22_01076-77, PYK22_01839-40, PYK22_02287-88), and lipo-oligosaccharide (*nodJI*, PYK22_00778 and PYK22_00785). These reflect the carbohydrate and polypeptide utilizing phenotype of the bacterium. *Pyrinomonas methylaliphatogenes* K22^T^ also possesses putative ABC transporters targeting amino acid cysteine, oligopeptides (*oppABCDF*, e.g. the PYK22_01277-281 cluster), and lipoproteins (*lolCDE*, PYK22_02373-4). Nitrogen assimilation is facilitated via an ammonia permease (PYK22_02853), the importation of oligopeptides by an *oppABCDF* ABC transporter system (similar to the system in *Salmonella typhimurium* [27]), and major facilitator superfamily nitrate/nitrite permeases (PYK22_00018 & PYK22_00946). Additionally, the *P. methylaliphatogenes* K22^T^ genome contained a cluster of genes *tonB-exbB-exbD-exbD* (PYK22_00991-94) associated with siderophore transport in some other acidobacterial species [26]. However, genes involved in siderophore synthesis, polyketide synthase, and nonribosomal peptide synthetase were not found, suggesting that it scavenges siderophores produced by other bacteria.

Based upon 16S rRNA gene sequence similarity, the most closely related and cultivated strain to *P. methylaliphatogenes* K22^T^ is *C. thermophilum* B^T^ [28] (Fig. 1)*.* The sequence similarity (~86 %) indicates that the two strains may belong to the same subdivision based on taxonomic sequence identity thresholds calculated for other prokaryotic taxa [29]. This phylogenetic dissimilarity between the two strains is also reflected in a comparison of the genomic content and the different metabolic modes of existence (chemoheterotrophic *P. methylaliphatogenes* K22^T^ vs. photoheterotrophic *C. thermophilum* B^T^) of the two strains. For example, the *C. thermophilum* B^T^ genome encodes for genes for chlorosomes, bacteriochlorophyll pigments *a* and *c* and a pigment protein complex for phototrophic growth, whereas no genes encoding for phototrophy were found in K22^T^. The *C. thermophilum* B^T^ genome also contained significantly more COGs (15 vs 50) related to signal transduction kinases (COG0515 and COG0642) than were encoded in *P. methylaliphatogenes* K22^T^. Conversely, *P. methylaliphatogenes* K22^T^ contained more genes related to amino acid utilization, such as amino acid transporters (COG0531) and amidohydrolases (COG1228), reflecting its ability to grow using proteinaceous media as the carbon and energy source. While both species possess carbohydrate-related metabolisms, the *P. methylaliphatogenes* K22^T^ genome encodes a much larger number of glycosyltransferases (COG0438 and COG0463) and beta-glucosidase-related glycosidases (COG1472) than that of *C. thermophilum* B^**T**^.

## Conclusions

*Acidobacteria* is one of the most widely-distributed bacterial phyla, particularly in soils [30–32]. Despite the wide distribution, the number of cultivated and sequenced representatives within most subdivisions within *Acidobacteria* remains low [33]. The sequencing and annotation of the *P. methylaliphatogenes* K22^T^ genome presented here links the phenotypic traits of *P. methylaliphatogenes* K22^T^ [4] with its genetic characteristics, and represents a step that will assist future studies describing the ecological and metabolic capabilities of this widespread phylum.

## Additional file

Additional file 1:
**Associated MIGS Record and Sequencing and Assembly Methodologies.** (DOCX 39 kb)

## Footnotes

1 Editor’s note: Although the name *Acidobacteria* is in common use at the phylum and class level, readers are advised that it appears on the list of rejected names. By definition, a rejected name must not be used to designate any taxon (Rule 23 a Note Note 4 (i)) at any rank.
