# Supplementary material for: Complete genome sequence of the thermophilic Acidobacteria, Pyrinomonas methylaliphatogenes type strain K22T
Source: Stand Genomic Sci. 2015 Nov 14;10:101. doi: 10.1186/s40793-015-0099-5 (PMC4644332; doi:10.1186/s40793-015-0099-5)
Supplement: Additional file 1: — Associated MIGS Record and Sequencing and Assembly Methodologies. (DOCX 39 kb) [file 40793_2015_99_MOESM1_ESM.docx]

# Associated MIGS Record

**Table S1.** Associated MIGS record

| **MIGS-ID** | field name | description |
| --- | --- | --- |
| **MIGS-1** | Submit to INSDC/Trace archives |  |
| **1.1** | PID | Gp0050834  PRJEB4906  CBXV000000000 |
| **1.2** | Trace Archive | ENA SRA |
| **MIGS-2** | MIGS CHECK LIST TYPE |  |
| **MIGS-3** | Project Name | The annotated genome of *Acidobacteria* species *Pyrinomonas methylaliphatogenes*, strain K22^T^ |
| **MIGS-4** | Geographic Location | Mount Ngauruhoe, New Zealand |
| **4.1** | Latitude | 39°09'19.6"S |
| **4.2** | Longitude | 175°38'03.1"E |
| **4.3** | Depth |  |
| **4.4** | Altitude |  |
| **MIGS-5** | Time of Sample collection | 2006 |
| **MIGS-6** | Habitat (EnvO) | Geothermal soil |
| **6.1** | temperature | 68 °C |
| **6.2** | pH | 6.9 |
| **6.3** | salinity |  |
| **6.4** | chlorophyll |  |
| **6.5** | conductivity |  |
|  |  |  |
| **6.6** | light intensity |  |
| **6.7** | dissolved organic carbon (DOC) |  |
| **6.8** | current |  |
| **6.9** | atmospheric data |  |
| **6.10** | density |  |
| **6.11** | alkalinity |  |
| **6.12** | dissolved oxygen |  |
| **6.13** | particulate organic carbon (POC) |  |
| **6.14** | phosphate |  |
| **6.15** | nitrate |  |
| **6.16** | sulfates |  |
| **6.17** | sulfides |  |
| **6.18** | primary production |  |
| **MIGS-7** | Subspecific genetic lineage | *Pyrinomonas methylaliphatogenes* K22 |
| **MIGS-9** | Number of replicons | 1 |
| **MIGS-10** | Extrachromosomal elements | 0 |
| **MIGS-11** | Estimated Size | 3795097 |
| **MIGS-12** | Reference for biomaterial or Genome report | [[1](#_ENREF_1),[2](#_ENREF_2)] |
| **MIGS-13** | Source material identifiers | DSM 25857; ICMP 18710 |
| **MIGS-14** | Known Pathogenicity | No |
|  |  |  |
| **MIGS-15** | Biotic Relationship | Free-living |
| **MIGS-16** | Specific Host |  |
| **MIGS-17** | Host specificity or range (taxid) |  |
| **MIGS-18** | Health status of Host |  |
| **MIGS-19** | Trophic Level | Heterotroph |
| **MIGS-22** | Relationship to Oxygen | Obligate aerobe |
| **MIGS-23** | Isolation and Growth conditions | [[1](#_ENREF_1)] |
| **MIGS-27** | Nucleic acid preparation | Q-Biogene FastDNA SPIN kit |
| **MIGS-28** | Library construction | 454 single-end, Illumina paired-end |
| **28.1** | Library size |  |
| **28.2** | Number of reads | 75215 (454 Titanium), 1196578 (Illumina) |
| **28.3** | vector |  |
| **MIGS-29** | Sequencing method | 454, Illumina |
| **MIGS-30** | Assembly |  |
| **30.1** | Assembly method | MIRA 4.0rc2 |
| **30.2** | estimated error rate |  |
| **30.3** | method of calculation |  |
| **MIGS-31** | Finishing strategy |  |
| **31.1** | Status | Permanent draft |
| **31.2** | coverage | 75 X |
| **31.3** | contigs | 16 |
| **MIGS-32** | Relevant SOPs |  |
| **MIGS-33** | Relevant e-resources |  |

**Supplementary material - Sequencing and assembly methodologies**

**Sequencing library preparation protocol**

**Single-end 454 library preparation.** A single-end 454 library was constructed according to the manufacturer's protocols of 454 GS FLX Titanium Rapid Library kits for GS Junior (Rev. June 2010) and GS Junior Titanium emPCR kit (Lib-L, Version April, 2010), which is as follow:

The input DNA was fragmented by nebulization. Sample DNA (500 ng) was suspended in 100 μl TE buffer. Nebulization buffer (500 μl) was added and the mixture was placed inside the nebulizer. Nitrogen gas at 30 psi (2.1 bar) was applied for 1 minute and 2.5 ml of PBI buffer was then added.

The nebulized DNA was purified using Qiagen MinElute PCR Purification kit. This was done by loading 750 μl nebulized DNA into into a single column and spun down (13,000 rpm, 15 seconds) and discard flow through. The process was repeated on the same column until all nebulized DNA have been processed, and an additional 1 minute of centrifugation was added to fully remove all flow-through. PE Buffer (750 μl) was added into the column, and then further spun down for another 1 minute, with the flow-through discarded. Repeated centrifugation was done by spinning down 1 minute, rotate the column 180º, and then centrifuge 1 minute. Purified DNA was eluted into a new tube with 16 μl of TE Buffer by centrifuging for 1 minute.

The ends of the purified nebulized DNA was repaired by mixing the DNA with 9 μl of End Repair mix (containing: 2.5 μl RL 10x Buffer, 2.5 μl RL ATP, 1 μl RL dNTP, 1 μl RL T4 Polymerase, 1 μl RL PNK, and 1 μl RL Taq Polymerase) then incubated in a thermal cycler with at 25 ºC for 20 min, 72 ºC for 20 min, and hold on 4 ºC. After the end-repair program, 1 μl of RL Adaptor was added and then further incubated at 25 ºC for 10 minutes.

Small fragments were removed by AMPure Bead. The beads were prepared by aliquoting  125 μl of AMPure beads and removing the supernatant after immobilizing the beads with a magnetic stand. The beads were then resuspended in 73 μl TE Buffer and 500 μl sizing solution. The DNA sample was added into the prepared bead solution and incubated at RT for 5 minutes. The supernatant was moved after pelleting the beads on a magnetic stand. TE buffer (100 μl) and sizing solution (500 μl) were added with 5 seconds of vortexing in between, then further incubate for 5 minutes, and supernatant was removed after pelleting the beads with the magnetic stand. The TE/sizing buffer washing step was repeated once. The beads were then washed twice with 1 ml freshly prepared 70% molecular grade ethanol by mixing with the beads, and then removing the supernatant while immobilising the beads with the magnetic stand. The beads were air-dried on the stand for 2 minutes. The DNA was extracted by adding 53 μl of TE buffer to the beads and then removing 51 μl of supernatant (containing the DNA) with the beads pelleted on a magnetic stand.

The resulting DNA were assessed by running 1 μl of sample on an HS Bioanalyzer chip using manufacturer's protocol (2100 Bioanalyzer, Agilent Technologies, Santa Clara, USA). The library was quantified using a TBS 380 Fluorometer against 8 serial dilutions of the RL Standard. The stock of the standards were set up by mixing 90 μl of 2.5 x 109 molecule/μl solution of the RL Standard with 90 μl of TE Buffer, then serial dilute the stock by transferring 120 μl of the more concentrated standard into 7 tubes containing 60 μl TE buffer. The DNA was quantified on the Blue channel with the standard value (Std Val) to 250 compared with the standards. An aliquot of the DNA library was diluted to a working stock of

1 x 107 molecules/μl, in TE buffer for the emulsion PCR (emPCR) process.

The emulsion oil was prepared by shaking at 28 Hz for 2 minutes. One ml aliquot of Mock amplification mix (made from mixing  250 μl of 5x Mock Amplification Mix with 1 ml of molecular biology grade water, to make a 1.25 ml of 1x working solution) was added into the emulsion oil.

A Live Amplification Mix was made containing 300 μl molecular biology grade water,

375 μl emPCR Additive, 195 μl 5x Amplification Mix, 58 μl Amplification Primer, 50 μl emPCR Enzyme Mix, and 2 μl PPiase.

A 1x x Capture Bead Wash Buffer TW was made by combining 100 μl of 10x Capture Bead Wash Buffer TW with 900 μl molecular biology grade water.

A well suspended DNA Capture Beads solution (230 μl per reaction) was spun down (10 seconds) rotated 180 degrees and spun down again, then the supernatant was discarded. The beads were then washed twice with 200 μl of the 1x Capture Bead Wash Buffer TW by repeating the centrifuge-rotate tube-centrifuge, and then remove the supernatant steps above.

The above purified and quantified DNA library were denatured by heating at 95°C for 2 minutes and hold on 4 °C. Based on the above quantification a suitable amount, i.e. 1-20 μl of DNA library was added to the beads. This was calculated by desired molecules per bead x 5 million beads divided by the library concentration (in molecules/µl). Thus with 2 desired molecules per bead as recommended by Roche, and with a library concentration of 2 million molecules per µl, 5 µl of library would be used.

After the addition of DNA library with the beads, 875 μl of Live Amplification Mix was added and the content was then transported to the emulsion mix prepared earlier and shaken again at 12 Hz for 5 minutes. The mixture was then placed into a 96 well plate at 100 μl per well. The amplification process was conducted in a thermal cycler with the following parameters:

1x 4 minutes at 94°C

50x 30 seconds at 94°C, 4.5 minutes at 58°C, 30 seconds at 68°C

1x 10°C on hold

Emulsion in the PCR product was broken via vacuum assisted emulsion breaking with the GS FLX Titanium emPCR Breaking Kit. Connecting to a vacuum source, the PCR products in the wells were aspirated by the 8-channel transfer pipette and the beads would accumulate in the attached 50 ml centrifuge tube. Wash the wells three times with 100 μl of isopropanol per well and then slowly aspirate an additional (approximate) 5 ml of isopropanol to collect any beads that may remain in the tubing. The bead containing-tube was topped up with isopropanol to a final volume of 40 ml before pelleting the beads by centrifuging at 930 x g for 5 minutes, then discarding the supernatant carefully by pouring. The remaining pellet was resuspended by 20 ml of Enhancing Fluid XT, and then spun down discard supernatant as the above. The pellet was then washed with 40 ml isopropanol, 35 ml isopropanol, 35 ml ethanol, 35 ml Enhancing Fluid XT, all following the step of vortex to mix, centrifuge to pellet, and pouring to discard supernatant. For the Enhancing Fluid XT step, approximately 2 ml of the supernatant in order to transfer the suspension into smaller microcentrifuge tube to better remove the little remaining supernatant in a stepwise process. Finally the bead pellet was rinsed twice with 1 ml of Enhancing Fluid XT following the spin-rotate-spin-discard method.

The Melt Solution was prepared by mixing 125 μl of NaOH (10 N) in 9.875 ml of molecular biology grade water. The tube of beads from above was washed twice by vortex mixing with 1 ml of the Melt Solution, incubate for 2 minutes at room temperature, then spin-rotate-spin and discard the supernatant, the washed three times with 1 ml of Annealing Buffer XT. The beads were then resuspended in 30 μl of Annealing Buffer XT and 12 μl of Enrichment Primer and incubated at  65°C for 5 minutes, and then promptly cool on ice for 2 minutes before washing with 1 ml of Enhancing Fluid XT with the spin-rotate-spin-discard method three times.

The DNA library beads were resuspended in 1 ml of Enhancing Fluid XT and mixed with 40 μl aliquot of the washed Enrichment Beads. The Enrichment Beads were washed twice by using magnetic stand and resuspending with 500 μl of Enhancing Fluid XT then removing the supernatant while the magnetic beads were immobilized. The DNA-Enrichment beads mixture was rotated in RT for 5 minutes. The supernatant was removed after magnetic pelleting for 3-5 minutes. The beads were washed with Enhancing Fluid XT repeatedly with magnetic bead immobilisation until white DNA beads are no longer being aspirated during the removal of supernatant. The pellet was then resuspended in 700 μl of Melt Solution. On the magnetic stand, transfer the supernatant containing the DNA Beads while the Enrichment Beads pellet below. This process of resuspension and transfer of DNA Beads was repeated once.

The DNA Beads were spun down and the supernatant was removed. The pellet was washed three times with 1 ml of Annealing Buffer XT following the spin-rotate-spin-discard method before resuspending the pellet with 60 μl  Annealing Buffer XT and 12 μl of Seq Primer. The mixture was incubated at 65°C for 5 minutes, and then promptly cool on ice for 2 minutes to anneal the Seq Primer. The DNA beads was washed three times with 1 ml of Annealing Buffer XT following the vortex, spin-rotate-spin-discard process. The beads were then used for 454 pyrosequencing.

**Paired-end Illumina library preparation**. The paired-end Illumina library was constructed using the Nextera XT DNA Sample Preparation kit (part number #15031942, revision C), according to the manufacturer's protocol. All addition of reagent should be pipetted to mix The input DNA was first tagmented (tagged and fragmented) with Nextera XT transposome by mixing 10 μl TD Buffer and 5 μl input DNA at 0.2 ng/μl (1 ng total) with 5 μl Amplicon Tagment Mix (ATM). The mixture was incubated at 55°C for 5 minutes, then cooled down to 10°C. Immediately after reaching 10°C, 5 μl NT Buffer was added into the mixture and incubated at room temperature for 5 minutes. The tagmented DNA were assessed by running 1 μl of sample on an HS Bioanalyzer chip using manufacturer's protocol (2100 Bioanalyzer, Agilent Technologies, Santa Clara, USA) .

For the PCR amplification step, 15 μl Nextera PCR Master Mix (NPM) was added into the tagmented DNA from the above process. Five μl of index 2 and index 1 primers were then added.

PCR was performed with the following parameters:

72°C for 3 minutes.

95°C for 30 seconds.

12 cycles of: 95°C for 10 seconds, 55°C for 30 seconds, and 72°C for 30 seconds.

72°C for 5 minutes.

Hold at 10°C .

The thermal cycler lid was heated.

The PCR products were cleaned up with AMPure XP beads. Suspended AMPure XP beads (30 μl) were added into (50 μl) PCR product. The mixture was incubated in RT for 5 minutes, and then placed on a magnetic stand for 2 minutes. With the tube on the stand, the supernatant was then removed and the beads were washed twice with 200 μl freshly prepared 80% ethanol for 30 seconds before carefully removing the supernatant. The remaining beads were air-dried for 15 minutes with the tube still on the stand. The sample was then removed from the stand and the beads were resuspended with 52.5 μl of Resuspension Buffer (RSB ) and incubated for 2 minutes. The sample was then placed back on the stand and the purified DNA solution was removed without disturbing the beads, and was stored in a clean tube.

The libraries were then normalized. First, a bead mixture was made (for 96 samples) by combining 4.4 ml Library Normalization Additives 1 (LNA ) with 800 μl resuspended Library Normalization Beads 1 (LNB1). Second, 45 μl of the bead mixture was added into each library well which contains 20 μl supernatant from the above DNA purification step. The mixture was shaken 1800 rpm for 30 minutes, then placed at a magnetic stand for 2 minutes. The supernatant was removed and discarded and the remaining beads were washed twice with 45 μl Library Normalization Wash 1 (LNW1) solution. During each wash the sample was shaken at 1800 shaking for 5 minutes and the supernatant was removed with a pipette and 2 minutes on the magnetic stand. Freshly prepared NaOH (30 μl 0.1 N) was added to the washed sample and shaken at 1800 rpm for 5 minutes to fully resuspend the beads. Supernatant (30 μl) containing the library was transferred, after placing the sample on magnetic stand, into a new container with 30 μl Library Normalization Storage (LNS1) solution.

The normalized libraries were then pooled. Aliquot of the pooled library (24 μl) was thoroughly mixed with  576 μl Hybridization Buffer (HT1) and incubated at 96°C for 2 minutes before placing immediately into an ice bath for 5 minutes. The diluted pooled library was then loaded into a MiSeq reagent cartridge (from MiSeq Reagent Kit v1) for sequencing

**Assembly.** The combined 454 (28.9 Mbp) and Illumina (301 Mbp) sequencing data were assembled together using the hybrid assembly capability of MIRA 4.0 rc4 with the following parameters: genome assembly in accurate de novo  mode (job = genome,denovo,accurate), disabling read name length check (mrnl=0), and use 8 CPU threads (GE:not=8) for assembly. The two types of library were declared in the assembly manifest file as: single-end 454 library (technology=454) and paired-end Illumina library (technology = solexa, segment_placement = ---> <---, template_size = 250 750). The template size range was derived from Bioanalyzer readings.

References

1. Crowe MA, Power JF, Morgan XC, Dunfield PF, Lagutin K, Rijpstra WI, Rijpstra IC, Sinninghe Damste JS, Houghton KM, Ryan JL and others. *Pyrinomonas methylaliphatogenes* gen. nov., sp. nov., a novel group 4 thermophilic member of the phylum *Acidobacteria* from geothermal soils. International Journal of Systematic and Evolutionary Microbiology 2014;64(1):220-227.

2. Stott MB, Crowe MA, Mountain BW, Smirnova AV, Hou S, Alam M, Dunfield PF. Isolation of novel bacteria, including a candidate division, from geothermal soils in New Zealand. Environmental Microbiology 2008;10(8):2030-2041.
